# The systemic inflammation response index (SIRI) predicts survival in advanced non-small cell lung cancer patients undergoing immunotherapy and the construction of a nomogram model

**DOI:** 10.3389/fimmu.2024.1516737

**Published:** 2024-12-24

**Authors:** Chunhan Tang, Min Zhang, Hongyuan Jia, Tianlei Wang, Hongwei Wu, Ke Xu, Tao Ren, Long Liang

**Affiliations:** ^1^ Clinical Medical College, Chengdu Medical College, Chengdu, Sichuan, China; ^2^ Department of Oncology, The First Affiliated Hospital of Chengdu Medical College, Chengdu, Sichuan, China; ^3^ Department of Oncology, Meishan Traditional Chinese Medicine Hospital, Meishan, Sichuan, China; ^4^ Department of Radiation Oncology, Sichuan Clinical Research Center for Cancer, Sichuan Cancer Hospital & Institute, Sichuan Cancer Center, Afliated Cancer Hospital of University of Electronic Science and Technology of China, Chengdu, China

**Keywords:** systemic inflammation response index, non-small cell lung cancer, immunotherapy, nomogram model, prognostic

## Abstract

**Background:**

Inflammation and immune evasion are associated with tumorigenesis and progression. The Systemic Inflammation Response Index (SIRI) has been proposed as a pre-treatment peripheral blood biomarker. This study aims to compare the relationship between SIRI, various serum biomarkers, and the prognosis of NSCLC patients before and after treatment.

**Methods:**

A retrospective study was conducted on advanced NSCLC patients treated with anti-PD-1 drugs from December 2018 to September 2021. Peripheral blood markers were measured pre- and post-treatment, and hazard ratios were calculated to assess the association between serum biomarkers and progression-free survival (PFS) and overall survival (OS). Kaplan-Meier curves and Cox proportional hazards models were employed for survival analysis. A nomogram model was built based on multivariate Cox proportional hazards regression analysis using the R survival package, with internal validation via the bootstrap method (1,000 resamples). Predictive performance was expressed using the concordance index (C-index), and calibration plots illustrated predictive accuracy.The application value of the model was evaluated by decision curve analysis (DCA).

**Results:**

Among 148 advanced NSCLC patients treated with PD-1 inhibitors, the median PFS was 12.9 months (range: 5.4–29.2 months), and the median OS was 19.9 months (range: 9.6–35.2 months). Univariate analysis identified pre- and post-treatment SIRI, mGRIm-Score, and PNI as independent prognostic factors for both PFS and OS (p < 0.05). Multivariate analysis demonstrated that high post-SIRI and post-mGRIm-Score independently predicted poor PFS (P < 0.001, P = 0.004) and OS (P = 0.048, P = 0.001). The C-index of the nomogram model for OS was 0.720 (95% CI: 0.693–0.747) and for PFS was 0.715 (95% CI: 0.690–0.740). Internal validation via bootstrap resampling (B = 1,000) showed good agreement between predicted and observed OS and PFS at 1, 2, and 3 years, as depicted by calibration plots.

**Conclusion:**

SIRI is an important independent predictor of early progression in advanced NSCLC patients treated with PD-1 inhibitors and may assist in identifying patients who will benefit from PD-1 inhibitors therapy in routine clinical practice.

## 1 Introduction

According to the American Cancer Society, lung cancer remains the leading cause of cancer-related deaths worldwide (1). Non-small-cell lung cancer (NSCLC) is the most prevalent type, accounting for 80–85% of lung cancer cases. Most NSCLC patients are diagnosed at an advanced stage, often losing the opportunity for curative surgery (2). Recently, immune checkpoint inhibitors (ICIs), particularly those targeting the PD-1/PD-L1 axis, have become the standard treatment for advanced NSCLC (3). These inhibitors work by disrupting the inhibitory signaling pathways between T cells an

d antigen-presenting cells, thereby enhancing the body’s natural anti-tumor immune response (4). However, not all patients respond favorably to immune therapy; 4–29% may even experience hyperprogression (5). This highlights the need to identify reliable biomarkers that predict which patients are likely to benefit from PD-1 inhibitor therapy.

Previous studies have shown that certain immune and inflammatory cells in the peripheral blood, such as neutrophils, monocytes, lymphocytes, and platelets, are involved in tumor invasion and metastasis and can be used as prognostic indicators (6). Various immune-inflammation-related parameters, such as the neutrophil-to-lymphocyte ratio (NLR), platelet-to-lymphocyte ratio (PLR), systemic immune-inflammation index (SII), and prognostic nutritional index (PNI), have been proposed to help identify subgroups of NSCLC patients more likely to benefit from ICIs (7–12). Among these, the systemic inflammation response index (SIRI), calculated as neutrophil count × monocyte/lymphocyte count, has been shown to better reflect the balance between immune response and inflammation in the body (13). Previous studies have demonstrated that higher pre-treatment SIRI predicts poorer survival in patients receiving chemoradiotherapy for advanced NSCLC (14). However, the prognostic value of SIRI in NSCLC patients treated with ICIs has not yet been fully explored.

This study aims to evaluate the prognostic value of SIRI, mGRIm-Score, and PNI in patients with advanced NSCLC treated with PD-1 inhibitors by analyzing laboratory data and survival outcomes.

## 2 Materials and methods

### 2.1 Patient cohorts

This retrospective study included 148 advanced NSCLC patients treated with ICIs at Sichuan Cancer Hospital between December 2018 and September 2021. Inclusion criteria were: (1) age ≥ 18 years, (2) pathologically confirmed advanced NSCLC (AJCC 8th edition), (3) treatment with anti-PD-1 monoclonal antibodies, and (4) completed data collection and follow-up. Patients with second malignancies, severe comorbidities, systemic inflammation, autoimmune diseases, or psychiatric disorders preventing treatment adherence were excluded. Additionally, 26 patients were excluded from mGRIm-Score analysis due to missing baseline LDH measurements.

### 2.2 Data collection and follow-up

Patient demographic and clinical characteristics, including gender, age, smoking and drinking history, tumor stage, and peripheral blood markers before and six weeks after treatment, were collected. The following formulas were used for biomarker calculation:

- SIRI = neutrophil count × monocyte/lymphocyte count
- NLR = neutrophil to Lymphocyte Ratio
- mGRIm-Score = 1 point each for NLR > 6, LDH > upper normal limit, or albumin < 3.5 g/dL, with scores categorized as low (< 2) or high (≥ 2)
- PNI =Serum Albumin (g/L) + 5 × Peripheral Blood Lymphocyte Count (×10⁹/L)

Based on published literature, we will use a cutoff value of 45 for PNI to explore progression-free survival and overall survival rates above and below this threshold (12). The differences in biomarker levels before and after treatment were calculated (Δ = post-treatment biomarker level/pre-treatment biomarker level), with Δ ≥ 1 considered a positive/stable change and Δ < 1 considered a negative change. Patients were followed up every two months through outpatient visits or telephone interviews, with a median follow-up duration of 11.9 months. Disease progression was assessed based on RECIST 1.1 criteria.

### 2.3 Statistical analysis

Data were analyzed using SPSS 21.0 and R 4.2.1 software. The Kolmogorov-Smirnov test was used to assess data normality. Normally distributed variables were compared using t-tests, while non-normally distributed variables were compared using the Mann-Whitney U test. Categorical data were compared using chi-square or Fisher’s exact tests. Kaplan-Meier survival curves and log-rank tests were used for survival analysis. Univariate and multivariate Cox proportional hazards models were applied to evaluate the relationship between biomarker levels and PFS/OS, with hazard ratios (HRs) and 95% confidence intervals (CIs) reported. ROC curves were plotted to assess the predictive ability of SIRI, and a nomogram model was constructed to visually represent the prognosis of NSCLC patients. Internal validation was performed using the bootstrap method with 1,000 resamples.

## 3 Results

### 3.1 Patient characteristics

**Figure 1:**
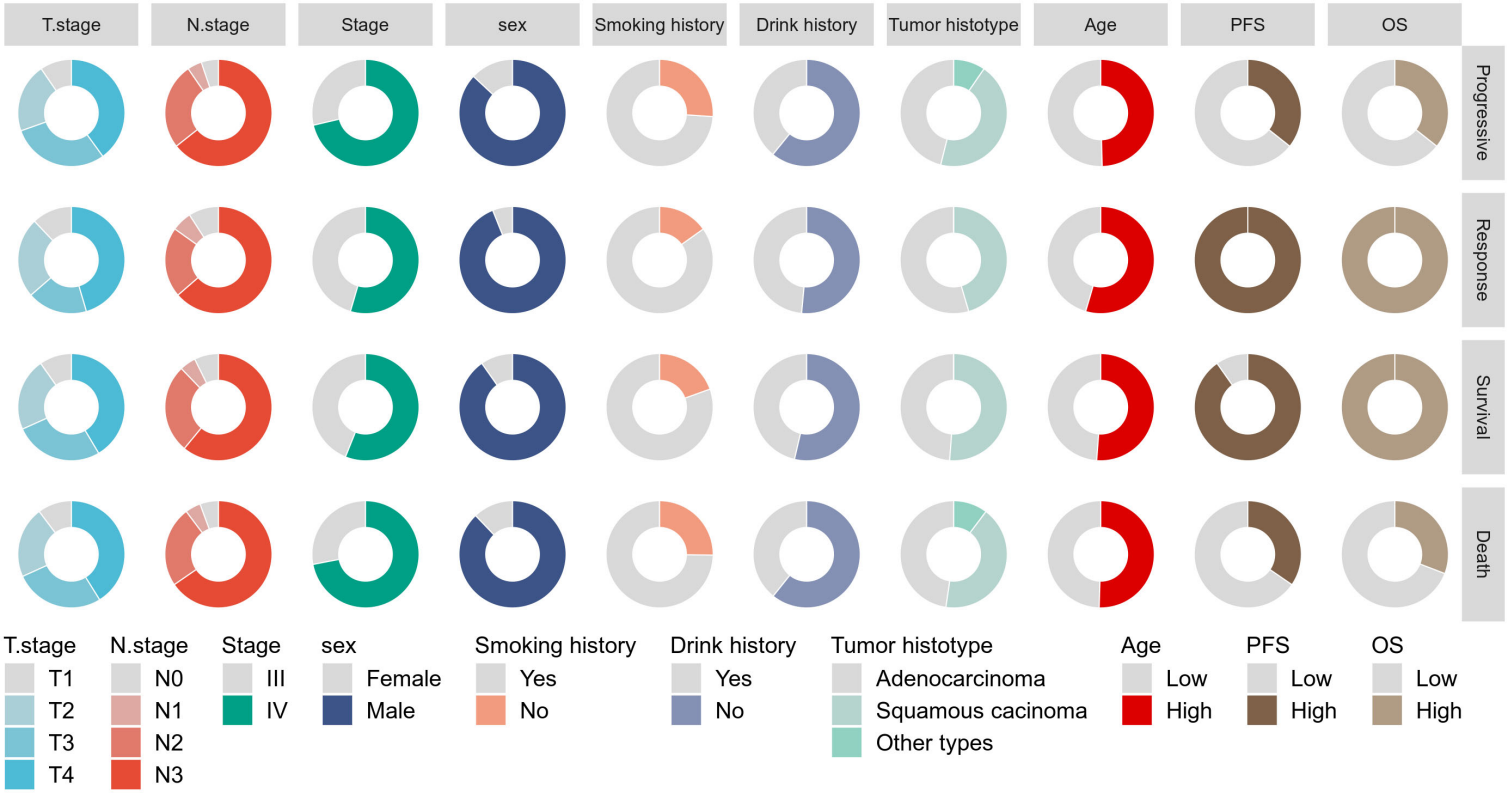
Display the baseline characteristics of patients using a pie chart.

This study included 148 patients with advanced NSCLC treated with PD-1 inhibitors. **Figure 1** summarizes the baseline characteristics of the entire cohort stratified by survival and disease response status. All patients received first- or second-line PD-1 therapy. The majority of patients had T4 stage (41.2%), N3 stage (64.2%), were male (88.5%), had a history of alcohol consumption (41.2%), were smokers (76.4%), had stage IV disease (67.6%), and were diagnosed with squamous cell carcinoma (48%). The median PFS for the entire cohort was 12.9 months (range: 5.4–29.2 months), and the median OS was 19.9 months (range: 9.6–35.3 months). The distribution of baseline variables across different survival and disease response status groups is shown in **Supplementary Table S1**. Notably, patients who had died exhibited significantly higher pre-mGRIm-Score, higher post-mGRIm-Score, higher pre-SIRI, higher post-SIRI, lower pre-PNI, and lower post-PNI compared to those who survived (p < 0.05). Patients with disease progression tended to have later disease stages, more squamous cell carcinoma histology, higher post-mGRIm-Score, higher pre-SIRI, higher post-SIRI, and higher post-PNI (p < 0.05).

**Figure 2:**
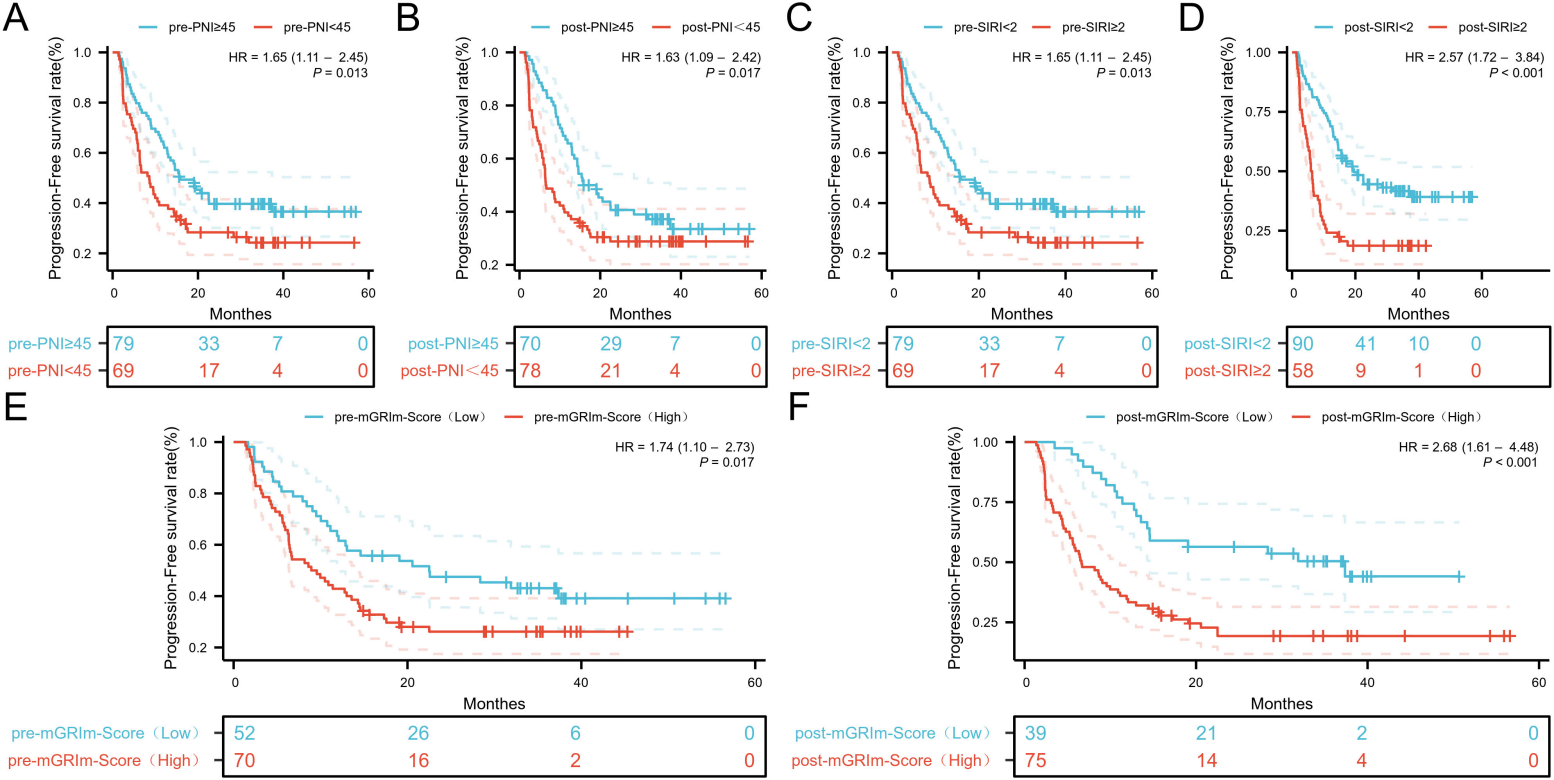
Kaplan-Meier estimates of PFS according to **(A)** pre-PNI, **(B)** post-PNI, **(C)** pre-SIRI, **(D)** post-SIRI, **(E)** pre-mGRIm-Score, **(F)** post-mGRIm-Score.

**Figure 3:**
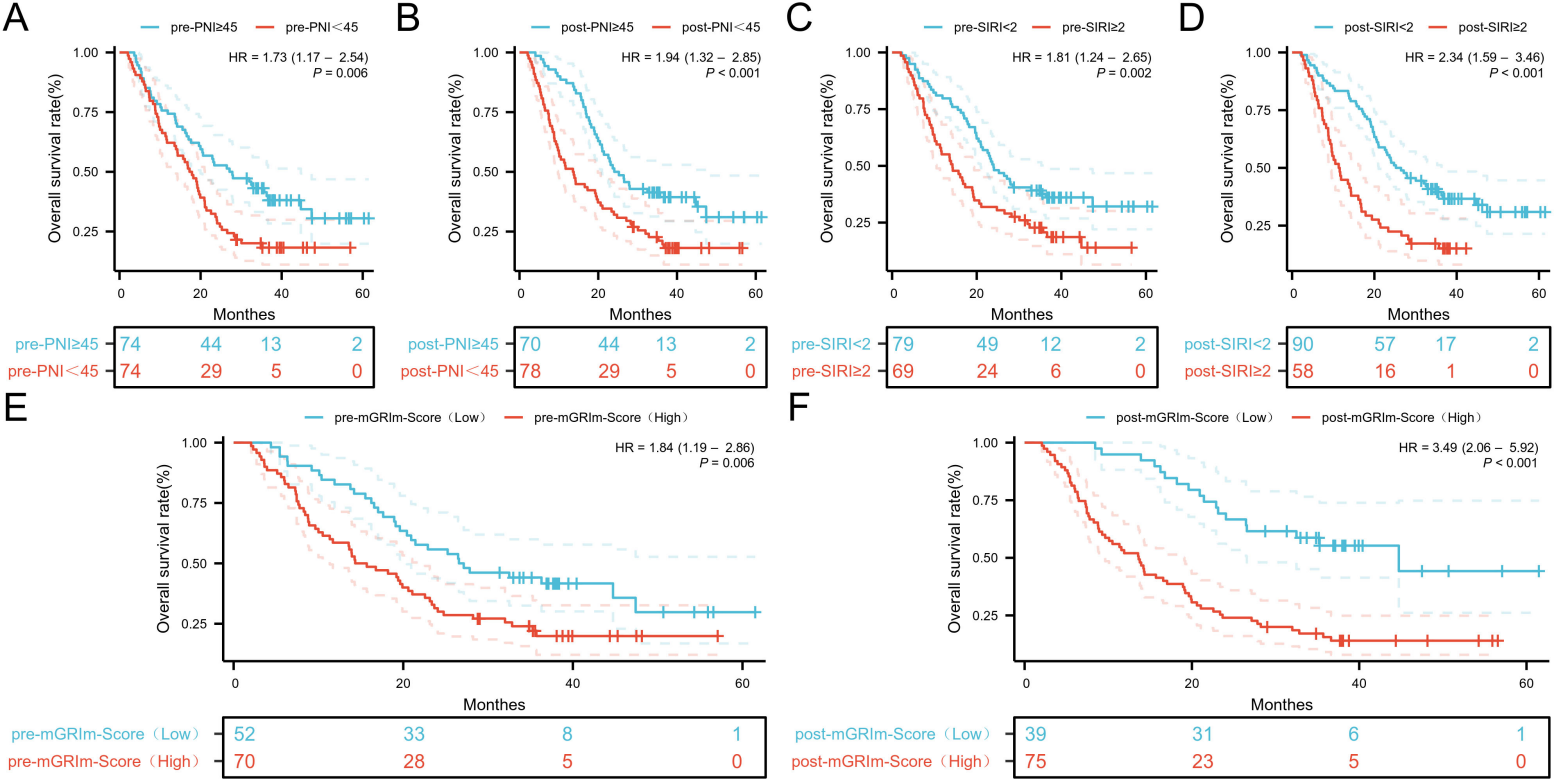
Kaplan-Meier estimates of OS according to **(A)** pre-PNI, **(B)** post-PNI, **(C)** pre-SIRI, **(D)** post-SIRI, **(E)** pre-mGRIm-Score, **(F)** post-mGRIm-Score.

ROC analysis was performed to determine the ability of the biomarker SIRI to predict patient prognosis and disease progression (**Supplementary Figure S1**; **Supplementary Figure S2**). SIRI, an index reflecting inflammation and immune status, had an optimal cut-off value of 2. This cut-off stratified SIRI into two categories: 90 patients (60.8%) had post-SIRI levels below the threshold, and 58 patients (39.1%) had post-SIRI levels above the threshold. Factors such as gender (P = 0.014), Pre-SIRI < 2 (P < 0.001), Pre-mGRIm-Score (P = 0.002), post-mGRIm-Score (P < 0.001), pre-PNI (P < 0.001), and post-PNI (P < 0.001) were significantly different from normal controls. Other demographic and clinical characteristics showed no statistical differences, as shown in **Table 1**. Survival analysis was performed based on pre- and post-treatment SIRI, mGRIm-Score, and PNI levels. Kaplan-Meier survival curves indicated significant differences in survival and disease progression rates among the groups (**Figures 2**, **3**). Patients with high pre-PNI, high post-PNI, low pre-SIRI, low post-SIRI, low pre-mGRIm-Score, and low post-mGRIm-Score had significantly higher survival rates than those with low pre-PNI, low post-PNI, high pre-SIRI, high post-SIRI, high pre-mGRIm-Score, and high post-mGRIm-Score (P-values: p = 0.006, p < 0.001, p = 0.002, p < 0.001, p = 0.006, p < 0.001, respectively, **Figure 3**).

**Table 1:** Demographic and clinical characteristics of cancer patients stratified by post-SIRI.

Characteristics	post-SIRI<2	post-SIRI≥2	P value
n	90	58	
T.stage, n (%)			0.337
T1	11 (7.4%)	4 (2.7%)	
T2	21 (14.2%)	11 (7.4%)	
T3	26 (17.6%)	14 (9.5%)	
T4	32 (21.6%)	29 (19.6%)	
N.stage, n (%)			0.926
N0	5 (3.4%)	4 (2.7%)	
N1	5 (3.4%)	2 (1.4%)	
N2	22 (14.9%)	15 (10.1%)	
N3	58 (39.2%)	37 (25%)	
Stage, n (%)			0.889
III	29 (19.7%)	19 (12.9%)	
IV	61 (41.5%)	38 (25.9%)	
sex, n (%)			0.014
Male	75 (50.7%)	56 (37.8%)	
Female	15 (10.1%)	2 (1.4%)	
Smoking history, n (%)			0.282
No	24 (16.2%)	11 (7.4%)	
Yes	66 (44.6%)	47 (31.8%)	
Drink history, n (%)			0.161
No	57 (38.5%)	30 (20.3%)	
Yes	33 (22.3%)	28 (18.9%)	
Tumor histotype, n (%)			0.061
Adenocarcinoma	45 (30.4%)	26 (17.6%)	
Squamous cacinoma	42 (28.4%)	24 (16.2%)	
Other types	3 (2%)	8 (5.4%)	
Pre-SIRI, n (%)			< 0.001
Pre-SIRI<2	59 (39.9%)	20 (13.5%)	
Pre-SIRI≥2	31 (20.9%)	38 (25.7%)	
Pre-mGRIm-Score, n (%)			0.002
Low	41 (33.6%)	11 (9%)	
High	36 (29.5%)	34 (27.9%)	
post-mGrim-Score, n (%)			< 0.001
Low	33 (28.9%)	6 (5.3%)	
High	35 (30.7%)	40 (35.1%)	
pre-PNI, n (%)			< 0.001
pre-PNI<45	43 (29.1%)	45 (30.4%)	
pre-PNI≥45	47 (31.8%)	13 (8.8%)	
post-PNI, n (%)			< 0.001
post-PNI<45	46 (31.1%)	48 (32.4%)	
post-PNI≥45	44 (29.7%)	10 (6.8%)	
PFS, median (IQR)	14.235 (9.685, 18.985)	6.13 (3.1025, 10.367)	< 0.001
OS, median (IQR)	25.85 (17.092, 37.725)	11.45 (7.2833, 21.342)	< 0.001

IQR interquartile range.

### 3.2 Univariate and multivariate cox regression analysis of SIRI with PFS and OS

For progression-free survival (PFS), time to progression was considered the dependent variable, and disease response status was treated as the event variable (1 for yes, 0 for no). For overall survival (OS), survival time was treated as the dependent variable, and death was treated as the event variable (1 for yes, 0 for no).

Patient characteristics, SIRI, and other factors were included as covariates in a Cox proportional hazards model for univariate analysis. With disease response as the event variable, Pre-SIRI, post-SIRI, Pre-mGRIm-Score, and post-mGRIm-Score were associated with short-term progression in NSCLC patients (P < 0.05). Multivariate Cox regression analysis revealed that age (HR: 0.372, 95% CI 0.202–0.685, p = 0.002) and post-mGRIm-Score (HR: 2.445, 95% CI 1.327–4.506, p = 0.004) were independent risk factors for disease response status in NSCLC patients. A post-SIRI level above the threshold (HR: 2.475, 95% CI 1.447–4.233, p < 0.001) was a predictor of poor PFS in NSCLC patients. **Table 1** summarizes the relevant data. When death was used as the event variable, Pre-SIRI, post-SIRI, pre-PNI, post-PNI, Pre-mGRIm-Score, and post-mGRIm-Score were associated with poor prognosis in NSCLC patients (P < 0.05). Multivariate Cox regression analysis revealed that post-SIRI (HR: 1.641, 95% CI 1.005–2.680, p = 0.048) and post-mGRIm-Score (HR: 2.731, 95% CI 1.484–5.024, p = 0.001) were independent risk factors for death in NSCLC patients, as shown in **Table 2**. In summary, using patients with post-SIRI < 2 as the reference group, those with post-SIRI ≥ 2 demonstrated a 2.48-fold increased risk of disease progression and a 1.64-fold increased risk of overall mortality. Similarly, when using patients with post-mGRIm-Score (low) as the reference group, those with post-mGRIm-Score (high) exhibited a 2.45-fold increased risk of disease progression and a 2.73-fold increased risk of overall mortality.

**Table 2:** Univariate and multivariate Cox regression analyses of factors associated with Progression-Free Survival and overall survival.

Characteristics	Total(N)	Univariateanalysis(PFS)		Multivariate analysis(PFS)		Univariate analysis(OS)		Multivariate analysis(OS)	
		Hazard ratio (95% CI)	P value	Hazard ratio (95% CI)	P value	Hazard ratio (95% CI)	P value	Hazard ratio (95% CI)	P value
T stage	148								
T1,T2	47	Reference				Reference			
T3.T4	101	1.033 (0.675 - 1.580)	0.883			0.950 (0.632 - 1.427)	0.804		
N stage	148								
N0-N2	53	Reference				Reference			
N3	95	1.105 (0.730 - 1.674)	0.637			1.154 (0.774 - 1.721)	0.481		
Stage	148								
III	48	Reference		Reference		Reference		Reference	
IV	100	1.564 (0.999 - 2.450)	0.051	1.231 (0.731 - 2.074)	0.434	1.448 (0.949 - 2.210)	0.086	1.050 (0.632 - 1.742)	0.851
Age	148								
Age<65	107	Reference		Reference		Reference			
Age≥65	41	0.678 (0.422 - 1.091)	0.109	0.372 (0.202 - 0.685)	0.002	1.094 (0.717 - 1.669)	0.677		
Pre-SIRI	148								
Pre-SIRI<2	79	Reference		Reference		Reference		Reference	
Pre-SIRI≥2	69	1.651 (1.111 - 2.452)	0.013	0.768 (0.435 - 1.356)	0.363	1.809 (1.235 - 2.651)	0.002	0.869 (0.503 - 1.500)	0.615
post-SIRI	148								
post-SIRII<2	90	Reference		Reference		Reference		Reference	
post-SIRII≥2	58	2.571 (1.721 - 3.842)	< 0.001	2.475 (1.447 - 4.233)	< 0.001	2.343 (1.589 - 3.456)	< 0.001	1.641 (1.005 - 2.680)	0.048
pre-PNI	148								
pre-PNI<45	88	Reference		Reference		Reference		Reference	
pre-PNI≥45	60	0.666 (0.441 - 1.004)	0.052	1.148 (0.632 - 2.083)	0.651	0.525 (0.350 - 0.787)	0.002	0.730 (0.408 - 1.307)	0.289
post-PNI	148								
post-PNI<45	94	Reference		Reference		Reference		Reference	
post-PNII≥45	54	0.583 (0.382 - 0.889)	0.012	0.842 (0.482 - 1.473)	0.547	0.520 (0.345 - 0.785)	0.002	0.859 (0.501 - 1.474)	0.582
Pre-mGRIm-Score	122								
Pre-mGRIm-Score (Low)	52	Reference		Reference		Reference		Reference	
Pre-mGRIm-Score (High)	70	1.735 (1.102 - 2.732)	0.017	1.516 (0.807 - 2.847)	0.196	1.843 (1.189 - 2.856)	0.006	1.149 (0.630 - 2.097)	0.650
post-mGrim-Score	114								
Post-mGRIm-Score (low)	39	Reference		Reference		Reference		Reference	
Post-mGRIm-Score (High)	75	2.684 (1.609 - 4.477)	< 0.001	2.445 (1.327 - 4.506)	0.004	3.492 (2.060 - 5.921)	< 0.001	2.731 (1.484 - 5.024)	0.001

SIRI, systemic inflammation response index; PNI, Prognostic Nutritional Index; mGrim-Score,modified-Gustave Roussy Immune Score; CI, confidence interval.

### 3.3 Establishment and validation of nomogram model

**Figure 4:**
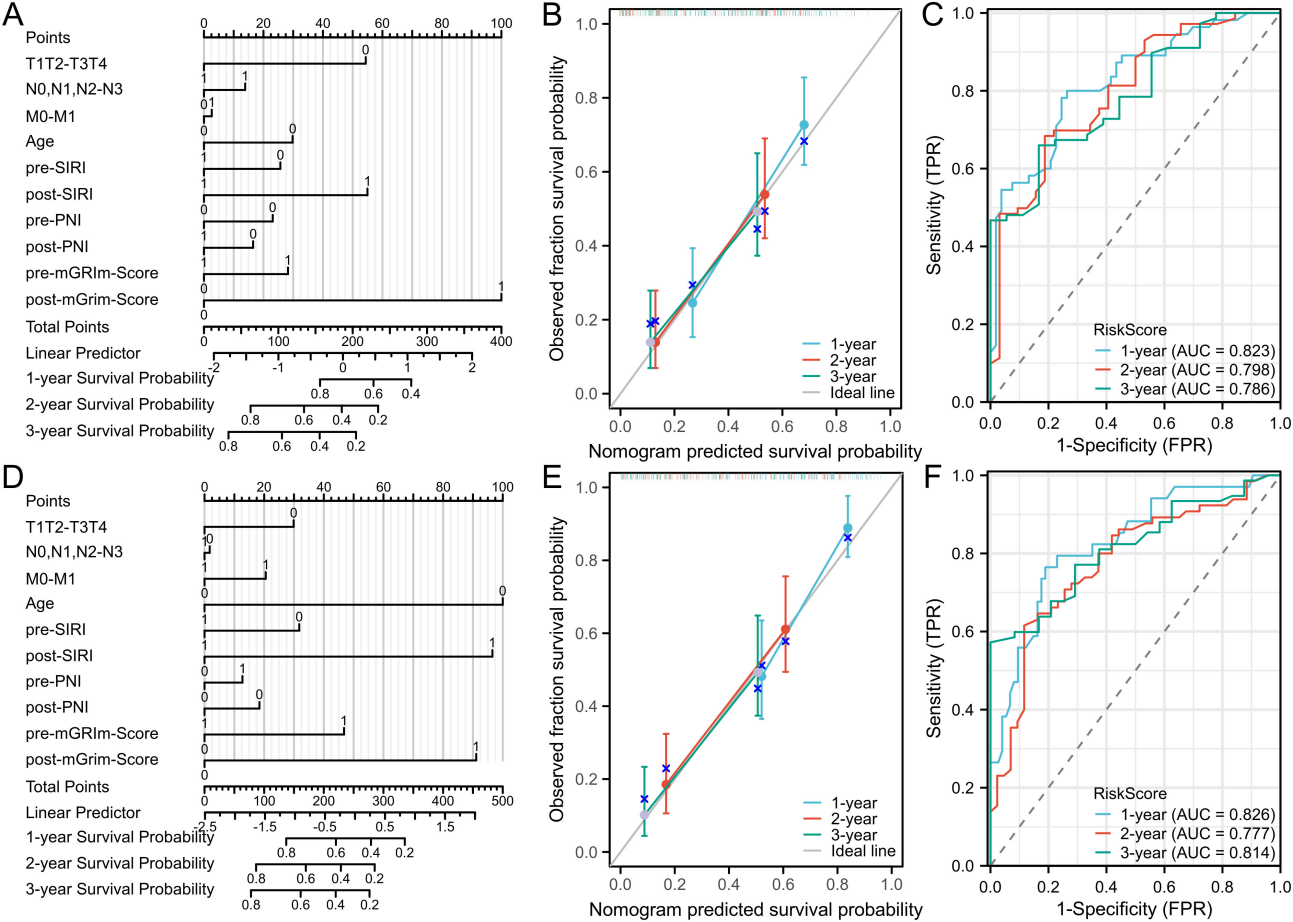
Nomogram and calibration curve for predicting PFS and OS of NSCLC patients and DCA curve of prediction model. **(A)** Nomogram model predicting 1-year, 2-year, and3-yearPFS; **(B)** calibration curves for 1-year, 2-year and 3-year disease Response rates; **(C)** 1-year PFS, 2-year PFS, and 3-year PFS clinical value DCA curves; **(D)** Nomogram model for OS; **(E)** calibration curves for 1-year, 2-year and 3-year survival rates; **(F)** 1-year, 2-year, and 3-year clinical value DCA curves.

To visually represent the prediction model results, a nomogram model was developed using R software based on statistically significant variables from the Cox regression analysis and TNM staging (tumor size, lymph node metastasis, distant metastasis, age, Pre-SIRI, post-SIRI, pre-PNI, post-PNI, Pre-mGRIm-Score, and post-mGRIm-Score). This model was used to assess the personalized prognosis of NSCLC patients receiving immunotherapy (**Figures 4A, D**). Scores corresponding to each indicator were summed to obtain a total score, which allowed for an intuitive estimation of 1-year, 2-year, and 3-year survival probabilities and progression risk. The higher the total score, the worse the predicted prognosis.

The nomogram model’s predictive performance was evaluated using the C-index and calibration curves. The results showed that the C-index for PFS prediction was 0.715 (95% CI: 0.690–0.740), and the C-index for OS prediction was 0.720 (95% CI: 0.693–0.747). Internal validation was conducted using the bootstrap resampling method (B = 1,000). Calibration curves, which compared predicted survival rates against actual survival rates, indicated good agreement between predicted and observed probabilities at 1, 2, and 3 years for both OS and PFS (**Figures 4B, E**). The model demonstrated good fit for PFS and OS. ROC curves were plotted based on independent predictors to assess the model’s accuracy in predicting 1-year, 2-year, and 3-year disease progression. The AUC for PFS was 0.823 (95% CI: 0.746–0.901), 0.798 (95% CI: 0.708–0.889), and 0.786 (95% CI: 0.682–0.890), respectively. The model exhibited good discriminatory ability (**Figure 4C**). For OS, the AUCs were 0.826 (95% CI: 0.741–0.911), 0.777 (95% CI: 0.688–0.867), and 0.814 (95% CI: 0.731–0.897), respectively, indicating similarly strong discrimination (**Figure 4F**). Decision curve analysis (DCA) was used to evaluate the model’s clinical utility, and the results showed a positive correlation between the threshold probability and the model’s net benefit when the threshold probability exceeded 0.05 (**Supplementary Figure S3**).

### 3.4 Relationship between changes in serum biomarkers and OS and PFS

**Figure 5:**
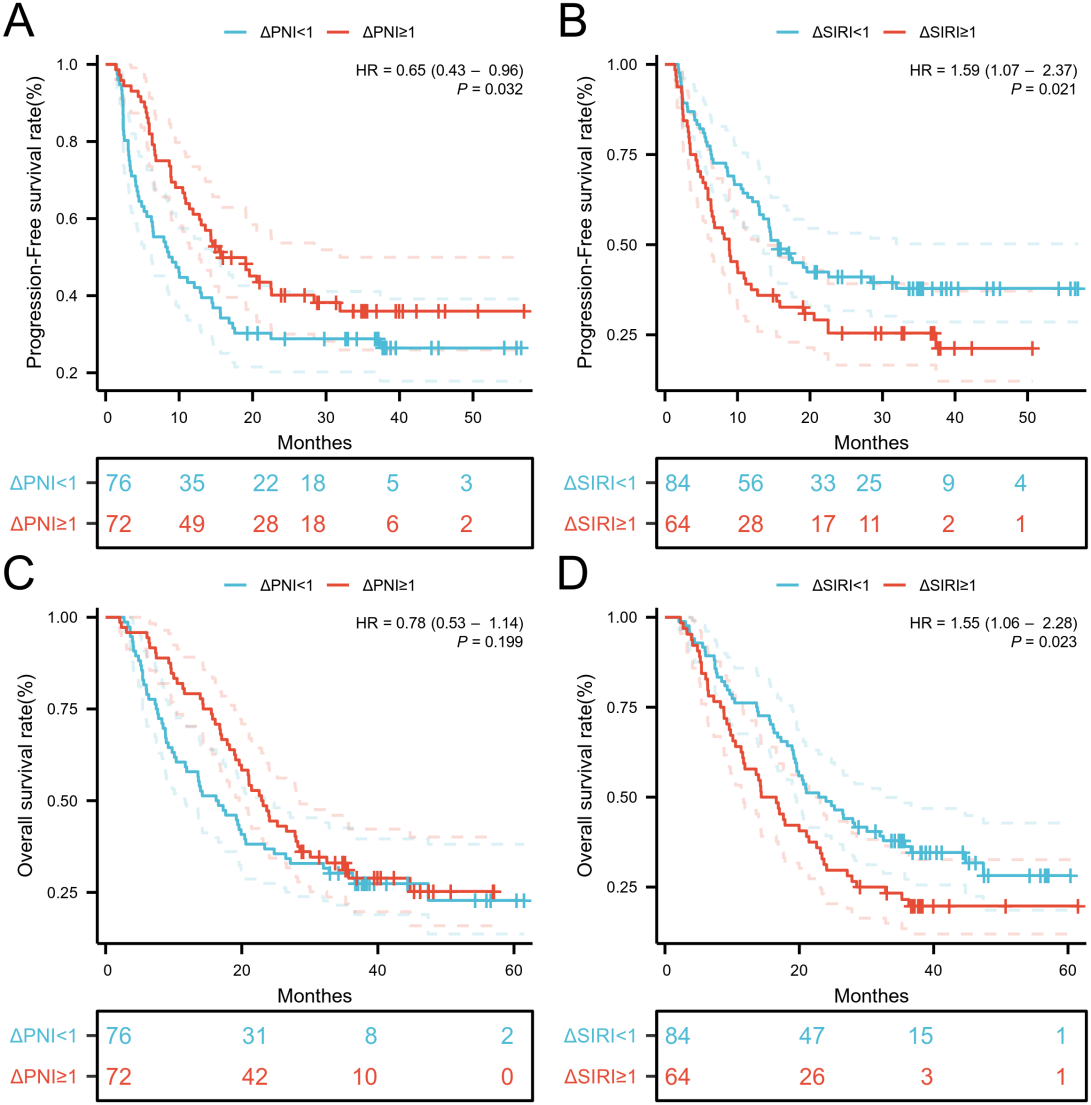
Kaplan-Meier estimates of PFS and OS according to **(A)** PFS of ΔPNI, **(B)** PFS of ΔPNI, **(C)** OS of ΔSIRI, **(D)** OS of ΔSIRI.

When the variation of SIRI was less than 1, the number of patients with death and disease progression was significantly lower, while the variation of PNI showed no obvious relationship with death or disease progression (**Supplementary Figure S4**). Kaplan-Meier curves and log-rank tests indicated that the low ΔSIRI group exhibited better prognosis compared to the high ΔSIRI group (**Figures 5B, D**). A high ΔPNI was associated with disease progression following immunotherapy, but it was not related to patient survival (**Figures 5A, C**).

## 4 Discussion

In recent years, immunotherapy has made remarkable progress in NSCLC, but the objective response rate in patients receiving immunotherapy remains around 20%, with a median response duration of approximately 13 months (15, 16). Studies such as KEYNOTE-021, KEYNOTE-189, IMpower-132, and CheckMate 227 have shown that combining immunotherapy with chemotherapy for advanced NSCLC improves ORR, PFS, and OS compared to chemotherapy alone (17–20). However, a significant proportion of NSCLC patients do not benefit from immunotherapy (21), making it essential to identify predictive biomarkers for those who may respond to treatment.

This study analyzed the relationship between inflammatory biomarkers and the PFS and OS of advanced NSCLC patients undergoing immunotherapy. The findings indicated that pre- and post-treatment levels of SIRI, mGRIm, and PNI were significantly associated with both PFS and OS (10, 12). Pre-treatment levels of mGRIm and PNI were consistent with previous studies in prognostic significance, while our multivariate analysis revealed that post-treatment SIRI and mGRIm-Score had higher specificity in predicting prognosis. Based on these results, we developed a nomogram model that showed high predictive accuracy and clinical utility, providing an important tool for assessing the prognosis of NSCLC patients receiving immunotherapy.

The association between SIRI and immunotherapy outcomes in NSCLC is reported here for the first time. Measuring systemic inflammatory indices at diagnosis may be a crucial consideration in clinical practice for advanced NSCLC. SIRI has already been shown to predict survival outcomes in several cancers, including pancreatic, liver, gastroesophageal junction adenocarcinoma, gastric, and renal cell carcinoma (22–26). Furthermore, SIRI can be used to dynamically monitor responses to immunotherapy. Advanced techniques such as multiparameter flow cytometry or single-cell RNA sequencing may further clarify the roles of neutrophils, monocytes, and lymphocytes in the blood, offering deeper insights into SIRI’s biological significance (27). Additionally, combining SIRI with other emerging blood biomarkers like circulating tumor DNA (ctDNA) could enhance prognostic predictions and therapeutic response assessments by reflecting both immune status and tumor genomic changes (28). This multi-layered biomarker combination may also be useful for identifying the phenomenon of “pseudoprogression” in immunotherapy. By combining SIRI with other biomarkers, clinicians can better differentiate between true progression and pseudoprogression, thereby optimizing treatment decisions (29).

SIRI, calculated using neutrophil, monocyte, and lymphocyte counts in peripheral blood, represents the balance between host immunity and inflammation. Neutrophils promote tumor metastasis and angiogenesis by secreting pro-inflammatory cytokines, reactive oxygen species (ROS), and neutrophil extracellular traps (NETs) (30). For instance, under certain conditions, neutrophils may exhibit anti-tumor characteristics, such as promoting tumor cell apoptosis or synergizing with T cells to exert effects (31). This finding complicates the role of neutrophils and raises new hypotheses that SIRI may not solely indicate a systemic pro-inflammatory state. Instead, the dynamic changes in SIRI could reflect the transition processes among different neutrophil subtypes, regulated by key signaling molecules in the microenvironment, such as TGF-β or IFN-γ (32). Monocytes differentiate into tumor-associated macrophages (TAMs), which promote tumor growth, migration, and angiogenesis (33). TAMs are generally classified into M1 (anti-tumor) and M2 (pro-tumor) subtypes, though recent studies suggest that TAMs display a spectrum of functional states rather than being strictly divided (34). Their role depends on signals from the tumor microenvironment, and they may shift between promoting and inhibiting tumor growth under certain conditions. This plasticity implies that monocyte counts might not always correlate with tumor progression but could reflect the adaptive behavior of macrophages in the tumor environment (35). Therefore, SIRI may indicate monocyte responses that could provide a therapeutic target, especially when combining immunotherapy with treatments aimed at reprogramming macrophages.

Lymphocytes, particularly CD8+ T cells, play a crucial role in anti-tumor immunity by recognizing and killing tumor cells and inducing apoptosis (36). B cells are also gaining recognition for their roles in forming tertiary lymphoid structures (TLS) within tumors, which are associated with improved prognoses in many cancers (37). These structures may enhance local immune responses and boost the efficacy of immune checkpoint inhibitors (ICIs). Consequently, changes in SIRI could reflect not only systemic immune status but also the formation of local immune structures like TLS (38). These physiological mechanisms partially explain SIRI’s predictive power in NSCLC patients undergoing immunotherapy.

The immune system’s role in lung cancer prognosis is vital. Chronic inflammation has been associated with metastasis in several cancers, including lung cancer (39). Although inflammation is necessary for activating the adaptive immune system, chronic inflammation can lead to immunosuppression, ultimately exhausting the immune system (40–42). Tumor-induced chronic inflammation alters peripheral blood cells, including macrophages, neutrophils, adipocytes, dendritic cells, and T, B, and NK cells, contributing to the tumor microenvironment (43). Inflammatory mediators like IL-2, IL-6, and PGE2 can initiate immunosuppression and are regulated by inflammatory feedback (44). Immune checkpoints, including PD-1, CTLA-4, and Fas, play critical roles in regulating immune responses. When engaged, these inhibitory receptors lead to immune tolerance through T cell exhaustion and apoptosis (45). SIRI, combining potent immune and inflammation indicators, captures the interactions between neutrophils, monocytes/macrophages, and lymphocytes within the tumor microenvironment.

This study compared various serum biomarkers before and after treatment and constructed a nomogram model to assess the prognostic value in non-small cell lung cancer (NSCLC) patients receiving immunotherapy. The model was evaluated using the C-index and calibration curves, and the results indicated that it demonstrated high predictive accuracy and clinical applicability. When integrated into electronic medical records, this model could provide significant clinical reference value for NSCLC prognosis assessment. However, several limitations should be acknowledged: as a retrospective analysis, the results may be influenced by factors such as small sample size, lack of external validation, and single-center data, which could introduce bias. Nevertheless, these findings lay a foundation for future prospective studies to validate these results.

## 5 Conclusion

SIRI is a readily accessible, cost-effective biomarker that can predict survival outcomes in advanced NSCLC patients undergoing PD-1 inhibitor therapy. The nomogram model developed in this study offers a reliable tool for clinicians to assess patient prognosis and tailor treatment strategies accordingly. Further studies are warranted to explore the broader application of SIRI in cancer immunotherapy and its potential integration into routine clinical practice.

## Data Availability

The raw data supporting the conclusions of this article will be made available by the authors, without undue reservation.

## Supplementary material

Supplementary Table 1 The distribution of background variables stratified by disease response status and survival status. SD,Standard Deviation

Supplementary Figure 1 ROC curves of the pre-SIRI and post-SIRI for predicting Disease progression occurrence.

Supplementary Figure 2 ROC curves of pre-SIRI and post-SIRI for predicting patient death occurrence.

Supplementary Figure 3 DCA curve of prediction model. **(A)** 1-year clinical value DCA curves of PFS, **(B)** 2-year clinical value DCA curves of PFS,**(C)** 3-year clinical value DCA curves of PFS, **(D)** 1-year clinical value DCA curves of OS,**(E)** 2-year clinical value DCA curves of OS,**(F)** 3-year clinical value DCA curves of OS

Supplementary Figure 4 A heatmap visualizes the changes in SIRI and PNI in populations with disease progression, responders, survivors, and non-survivors.
