# Supplementary material for: The systemic inflammation response index (SIRI) predicts survival in advanced non-small cell lung cancer patients undergoing immunotherapy and the construction of a nomogram model
Source: Front Immunol. 2024 Dec 24;15:1516737. doi: 10.3389/fimmu.2024.1516737 (PMC11703897; doi:10.3389/fimmu.2024.1516737)
Supplement: Supplementary Table 1 — The distribution of background variables stratified by disease response status and survival status. SD,Standard Deviation [file Table1.docx]

Table S1 The distribution of background variables stratified by disease response status and survival status

| Characteristics |  | All patients  (N=148) | Survival（N=41） | Death（N=107） | P value | Response（N=49） | Progressive（N=99） | P value |
| --- | --- | --- | --- | --- | --- | --- | --- | --- |
| T.stage, n (%) |  |  |  |  | 1.000 |  |  | 0.147 |
| T1 |  | 15 (10.1%) | 4 (2.7%) | 11 (7.4%) |  | 4 (2.7%) | 11 (7.4%) |  |
| T2 |  | 32 (21.6%) | 9 (6.1%) | 23 (15.5%) |  | 12 (8.1%) | 20 (13.5%) |  |
| T3 |  | 40 (27%) | 11 (7.4%) | 29 (19.6%) |  | 8 (5.4%) | 32 (21.6%) |  |
| T4 |  | 61 (41.2%) | 17 (11.5%) | 44 (29.7%) |  | 25 (16.9%) | 36 (24.3%) |  |
| N.stage, n (%) |  |  |  |  | 0.958 |  |  | 0.451 |
| N0 |  | 9 (6.1%) | 3 (2%) | 6 (4.1%) |  | 4 (2.7%) | 5 (3.4%) |  |
| N1 |  | 7 (4.7%) | 2 (1.4%) | 5 (3.4%) |  | 4 (2.7%) | 3 (2%) |  |
| N2 |  | 37 (25%) | 11 (7.4%) | 26 (17.6%) |  | 11 (7.4%) | 26 (17.6%) |  |
| N3 |  | 95(64.2%) | 25 (16.9%) | 70 (47.3%) |  | 30 (20.3%) | 65 (43.9%) |  |
| Stage, n (%) |  |  |  |  | 0.065 |  |  | 0.023 |
| III |  | 48 (32.4%) | 18 (12.2%) | 30 (20.3%) |  | 22 (14.9%) | 26 (17.6%) |  |
| IV |  | 100 (67.6%) | 23 (15.5%) | 77 (52%) |  | 27 (18.2%) | 73 (49.3%) |  |
| sex, n (%) |  |  |  |  | 0.904 |  |  | 0.839 |
| Male |  | 131 (88.5%) | 37 (25%) | 94 (63.5%) |  | 43 (29.1%) | 88 (59.5%) |  |
| Female |  | 17 (11.5%) | 4 (2.7%) | 13 (8.8%) |  | 6 (4.1%) | 11 (7.4%) |  |
| Smoking history,n(%) |  |  |  |  | 0.464 |  |  | 0.469 |
| Yes |  | 113 (76.4%) | 33 (22.3%) | 80 (54.1%) |  | 36 (24.3%) | 78 (52.7%) |  |
| No |  | 35 (23.6%) | 8 (5.4%) | 27 (18.2%) |  | 13 (8.8%) | 21 (14.2%) |  |
| Alcohol history,n(%) |  |  |  |  | 0.433 |  |  | 0.584 |
| Yes |  | 61 (41.2%) | 19 (12.8%) | 42 (28.4%) |  | 18 (12.2%) | 41 (27.7%) |  |
| No |  | 87 (58.8%) | 22 (14.9%) | 65 (43.9%) |  | 31 (20.9%) | 58 (39.2%) |  |
| Tumor histotype,n(%) |  |  |  |  | 0.092 |  |  | 0.022 |
| Adenocarcinoma |  | 71 (48%) | 20 (13.5%) | 51 (34.5%) |  | 29 (19.6%) | 42 (28.4%) |  |
| Squamous cacinoma |  | 66 (44.6%) | 21 (14.2%) | 45 (30.4%) |  | 20 (13.5%) | 46 (31.1%) |  |
| Other tyoes |  | 11 (7.4%) | 0 (0%) | 11 (7.4%) |  | 0 (0%) | 11 (7.4%) |  |
| pre-mGRIm-Score, n (%) |  |  |  |  | 0.040 |  |  | 0.079 |
| Low |  | 52 (42.6%) | 20 (16.4%) | 32 (26.2%) |  | 22 (18%) | 30 (24.6%) |  |
| High |  | 70 (57.4%) | 15 (12.3%) | 55 (45.1%) |  | 19 (15.6%) | 51 (41.8%) |  |
| post-mGrim-Score, n (%) |  |  |  |  | < 0.001 |  |  | 0.003 |
| Low |  | 39 (34.2%) | 21 (18.4%) | 18 (15.8%) |  | 19 (16.7%) | 20 (17.5%) |  |
| High |  | 75 (65.8%) | 11 (9.6%) | 64 (56.1%) |  | 16 (14%) | 59 (51.8%) |  |
| pre-SIRI, median (IQR) |  | 1.87 (1.055, 3.0725) | 1.44 (0.85, 2.14) | 2.04 (1.13, 3.19) | 0.021 | 1.64 (0.85, 2.62) | 2.03 (1.15, 3.235) | 0.028 |
| post-SIRI, median (IQR) |  | 1.51 (0.7875, 3.2875) | 0.95 (0.64, 1.71) | 1.85 (1.03, 3.375) | 0.004 | 1.25 (0.67, 1.74) | 1.95 (0.925, 3.385) | 0.008 |
| pre-PNI, median (IQR) |  | 43.2 (39.763, 47.525) | 45.7 (41.55, 49.5) | 42.45 (39.025, 47.075) | 0.009 | 44.95 (39.95, 49.15) | 42.95 (39.25, 47.375) | 0.137 |
| post-PNI, mean ± sd |  | 43.026 ± 5.5834 | 45.774 ± 5.3472 | 41.973 ± 5.3294 | < 0.001 | 44.759 ± 5.7879 | 42.169 ± 5.3018 | 0.007 |
| Age,n(%), mean ± sd |  | 59.676 ± 8.7312 | 59.293 ± 9.4134 | 59.822 ± 8.4973 | 0.742 | 61.041 ± 9.4184 | 59 ± 8.3373 | 0.182 |
| BMI, median (IQR) |  | 22.438 (20.747, 24.401) | 22.222 (20.69, 24.447) | 22.583 (20.761, 24.304) | 0.916 | 22.432 (20.658, 23.765) | 22.444 (20.791, 24.499) | 0.564 |
| PFS, median (IQR) |  | 12.933 (5.45, 29.225) | 37 (33, 39.933) | 8.8667 (4.3333, 15.733) | < 0.001 | 15.6 (6.2333, 35.167) | 12.767 (5.1, 24.433) | 0.404 |
| OS, median (IQR) |  | 19.933 (9.6167, 35.267) | 38.367 (35.567, 46.233) | 14.267 (7.5333, 21.25) | < 0.001 | 19.9 (10, 35.567) | 19.967 (9.6, 34.533) | 0.697 |

SD,Standard Deviation

.
