# Supplementary figures and images for: The systemic inflammation response index (SIRI) predicts survival in advanced non-small cell lung cancer patients undergoing immunotherapy and the construction of a nomogram model
Source: Front Immunol. 2024 Dec 24;15:1516737. doi: 10.3389/fimmu.2024.1516737 (PMC11703897; doi:10.3389/fimmu.2024.1516737)

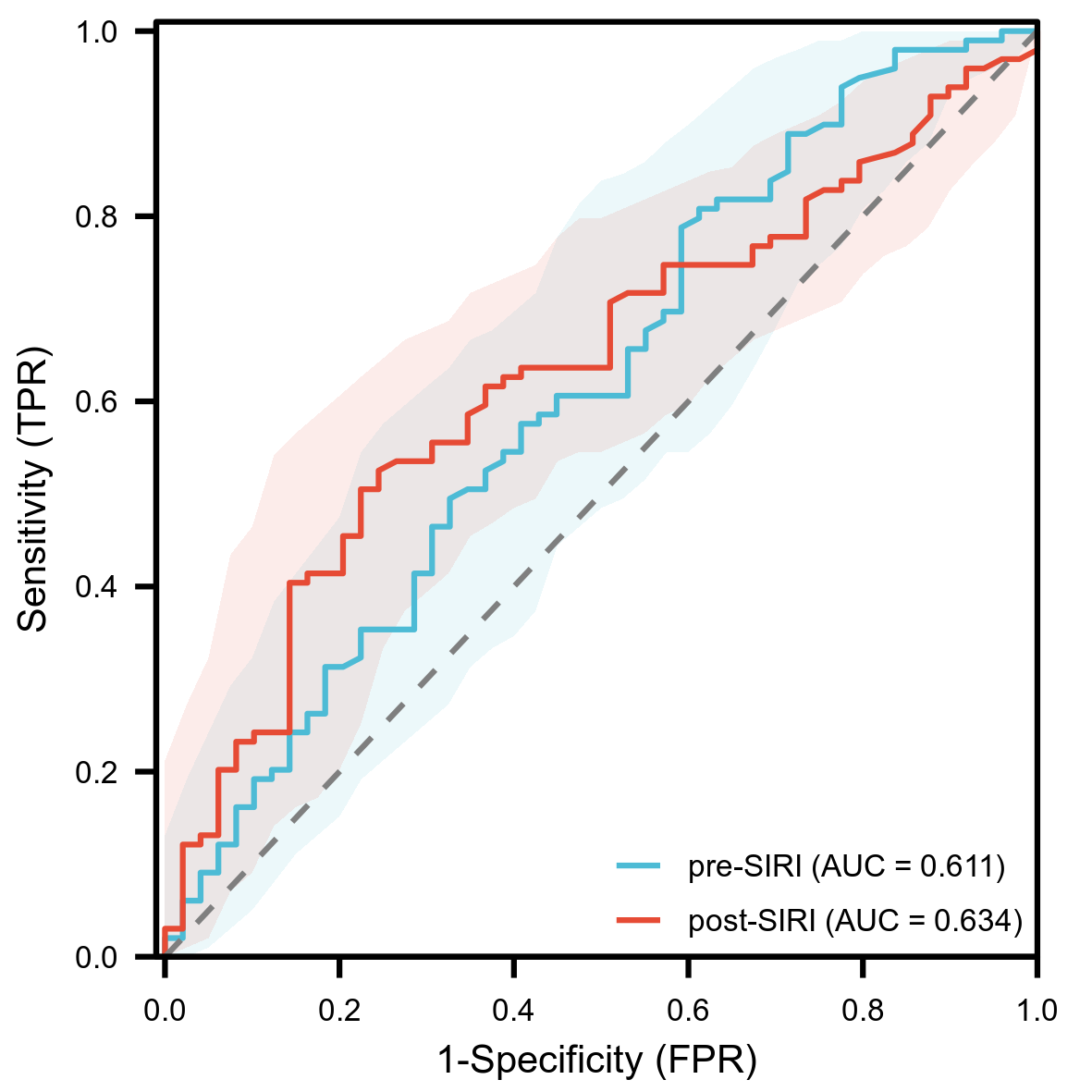

Supplement: Supplementary Figure 1 — ROC curves of the pre-SIRI and post-SIRI for predicting Disease progression occurrence. [file Image1.tiff]

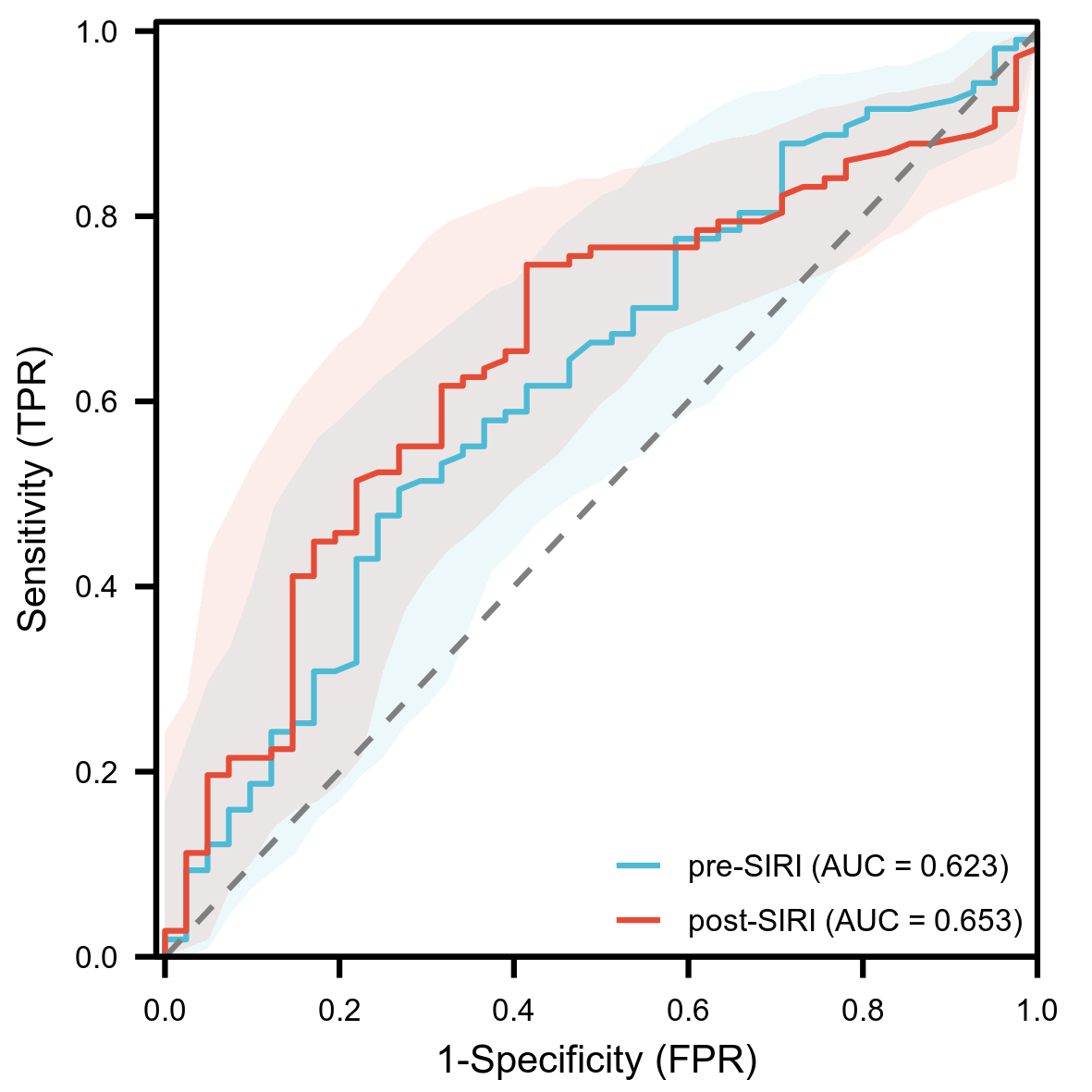

Supplement: Supplementary Figure 2 — ROC curves of pre-SIRI and post-SIRI for predicting patient death occurrence. [file Image2.tiff]

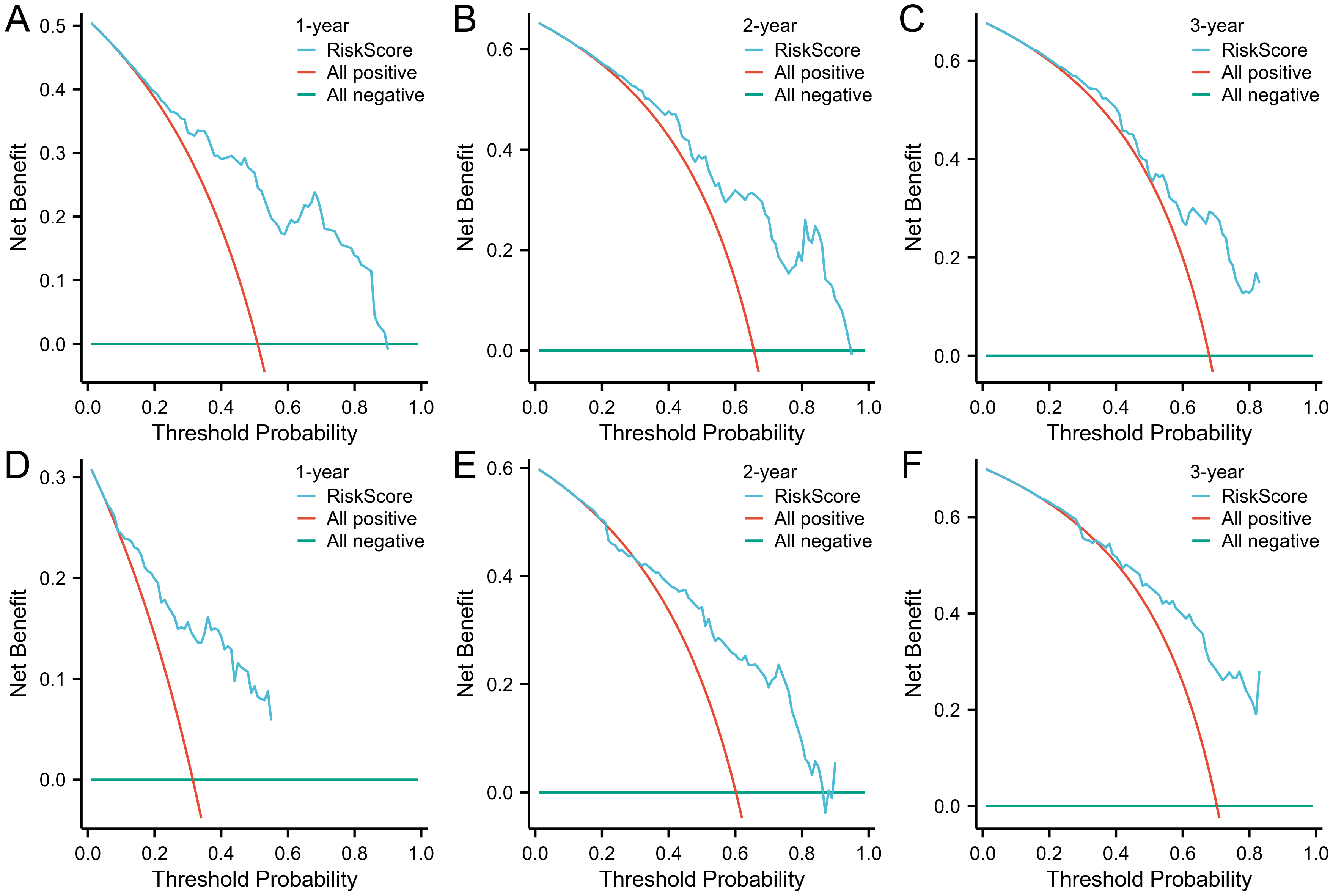

Supplement: Supplementary Figure 3 — DCA curve of prediction model. (A) 1-year clinical value DCA curves of PFS, (B) 2-year clinical value DCA curves of PFS,(C) 3-year clinical value DCA curves of PFS, (D) 1-year clinical value DCA curves of OS,(E) 2-year clinical value DCA curves of OS,(F) 3-year clinical value DCA curves of OS [file Image3.tiff]

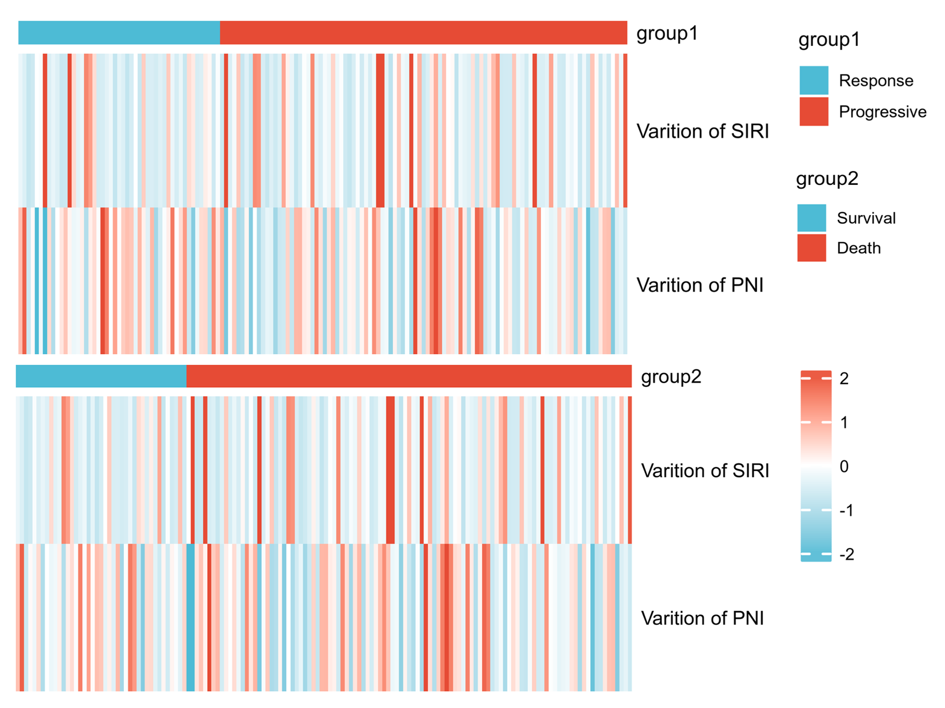

Supplement: Supplementary Figure 4 — A heatmap visualizes the changes in SIRI and PNI in populations with disease progression, responders, survivors, and non-survivors. [file Image4.tif]
